# Deep Recyclable Trash Sorting Using Integrated Parallel Attention

**DOI:** 10.3390/s24196434

**Published:** 2024-10-04

**Authors:** Hualing Lin, Xue Zhang, Junchen Yu, Ji Xiang, Hui-Liang Shen

**Affiliations:** 1 College of Information Science and Electronic Engineering, Zhejiang University, Hangzhou 310027, China; 22360394@zju.edu.cn (H.L.); zxue2019@zju.edu.cn (X.Z.); yujunchen@zju.edu.cn (J.Y.); 2 College of Electrical Engineering, Zhejiang University, Hangzhou 310027, China; jxiang@zju.edu.cn; 3 Huzhou Institute, Zhejiang University, Hangzhou 313000, China

**Keywords:** recyclable trash sorting, convolutional neural network, integrated parallel attention module

## Abstract

Sorting recyclable trash is critical to reducing energy consumption and mitigating environmental pollution. Currently, trash sorting heavily relies on manpower. Computer vision technology enables automated trash sorting. However, existing trash image classification datasets contain a large number of images without backgrounds. Moreover, the models are vulnerable to background interference when categorizing images with complex backgrounds. In this work, we provide a recyclable trash dataset that supports model training and design a model specifically for trash sorting. Firstly, we introduce the TrashIVL dataset, an image dataset for recyclable trash sorting encompassing five classes (TrashIVL-5). All images are collected from public trash datasets, and the original images were captured by RGB imaging sensors, containing trash items with real-life backgrounds. To achieve refined recycling and improve sorting efficiency, the TrashIVL dataset can be further categorized into 12 classes (TrashIVL-12). Secondly, we propose the integrated parallel attention module (IPAM). Considering the susceptibility of sensor-based systems to background interference in real-world trash sorting scenarios, our IPAM is specifically designed to focus on the essential features of trash images from both channel and spatial perspectives. It can be inserted into convolutional neural networks (CNNs) as a plug-and-play module. We have constructed a recyclable trash sorting network building upon the IPAM, which produces an acuracy of 97.42% on TrashIVL-5 and 94.08% on TrashIVL-12. Our work is an effective attempt of computer vision in recyclable trash sorting. It makes a positive contribution to environmental protection and sustainable development.

## 1. Introduction

In recent years, trash generation has continued to increase in line with economic and social progress [1]. Accordingly, waste management has become a key factor in sustained economic and social development. Manual trash sorting methods are labor-intensive and time-consuming [2]. Therefore, the automated sorting and recycling of trash has become an urgent issue.

With the rapid advancement of sensing, artificial intelligence, and computer vision technology, the automatic identification of trash from images as a replacement for manual sorting has become increasingly feasible. In industrial applications, most trash images are acquired using RGB imaging sensors. For specific trash such as plastics and organics, multispectral imaging sensors are sometimes employed for fine-level classification [3]. A convolutional neural network (CNN) is a deep learning algorithm in the field of image processing and computer vision [4,5]. The works [6,7,8] apply CNNs to trash sorting, achieving promising results in classification. Works like [9,10] introduce attention mechanisms to improve the model’s focus on key areas of the image. These methods enhance the model’s ability to capture salient features, but they do not fully consider the contribution of individual weights in the attention mechanism. This limitation may result in the suboptimal suppression of less important channels or pixels. Our approach addresses this gap by incorporating weight contribution factors into the attention mechanism, which allows the model to adaptively adjust feature importance and enhance trash sorting accuracy.

Along the development of computer vision technology, the demand for image datasets is gradually rising [11]. However, there are few public and authoritative datasets in the recyclable trash sorting field. Hence, it is imperative to develop a representative trash image dataset to assess and advance the development of trash sorting methods.

In this work, we introduce the TrashIVL dataset dedicated to sorting recyclable trash. The dataset consists of five categories of recyclable trash (TrashIVL-5), including clothes, plastic, paper, metal, and glass. The images are collected from public trash datasets. When building TrashIVL, we selectively screen out the trash images without background. In addition, we subdivide TrashIVL into 12 classes (TrashIVL-12) to improve the recycling efficiency and to achieve fine-sorting of recycling.

The background of trash images in TrashIVL varies, including grass, gravel roads, fallen leaves, etc. These backgrounds may interfere with the classification decisions of the CNNs. Focusing on this problem, we propose an integrated parallel attention module (IPAM) to enhance the trash sorting capability of CNNs. IPAM is a normalization-based attention module comprising a channel attention module (CAM) and a spatial attention module (SAM). The normalized weights obtained store scaling factors, which enables the adjustment of normalized features through scaling. These scaling factors allow the model to adaptively learn the importance of different features, which enhances the representational capacity of the model and improve the accuracy of trash sorting. To summarize, the main contributions of this work are as follows:

- We introduce a recyclable trash image dataset named TrashIVL. It excludes images without background found in public datasets, ensuring that the image backgrounds are more representative of real-life scenarios.
- We propose the integrated parallel attention module (IPAM) to improve the sorting ability of CNNs. IPAM acts as a plug-and-play module that can be inserted into different CNNs. It leverages the normalized weights and parallel connection of CAM and SAM.
- We construct a recyclable trash sorting network based on IPAM. When comparing different deep learning models of trash sorting, our network achieves the best classification performance.

## 2. Related Work

In this section, we first review the application of CNNs in trash sorting, and then introduce common trash image classification datasets.

### 2.1. Trash Sorting

With the development of deep learning, CNNs have shown their potential in the field of trash sorting. AlexNet [6] has been employed to categorize recyclable trash from landfill trash objects. DenseNet169 with transfer learning [7] has been used for classifying individual recyclable trash images. The optimized DenseNet121 [8] has leveraged genetic algorithms to optimize the fully connected layers and classify the TrashNet dataset.

The attention mechanism enables CNNs to identify and process key information in images more accurately by simulating human visual attention [12]. For example, AM-b Xception [9] fuses the multi-branch Xception network with an attention mechanism, which has been used to classify recyclable trash. Similarly, EfficientNet-B2 [10] has been applied to classify different types of trash: bio, glass, metal and plastic, non-recyclable, other, paper, and unknown. These methods dynamically select important regions in the image to focus on through an attention module. However, these attention mechanisms focus on important features by introducing additional convolutional and fully connected layers, overlooking the fact that the weights themselves can inherently represent the importance of features. In contrast, our approach leverages the inherent contribution of weights to adaptively adjust feature importance without the need for additional layers, thereby improving both the efficiency and accuracy of trash sorting.

### 2.2. Trash Image Classification Datasets

At present, the number of public trash image classification datasets is still limited. Table 1 shows the comparison of our TrashIVL and other trash image classification datasets. TrashNet [13] has been widely used, while its limited classes hinder its ability to provide finer recycling distinctions. Moreover, the presence of overlapping samples and unrefinable classes within the dataset restrict its representativeness in practical applications. In the Drinking waste classification [14], many of the images depict the same object captured from various angles, potentially leading to data redundancy. Additionally, several public trash datasets contain a significant number of images without backgrounds, deviating from real-life scenarios (e.g., Waste pictures [15], Trashbox [16], Garbage dataset [17], Kaggle garbage classification dataset [18], Huaweiyun garbage classify learning [19], and Garbage265 [20]).

## 3. Proposed Dataset—TrashIVL

In this section, we introduce our proposed TrashIVL dataset. First, we describe the source of TrashIVL. Next, we show how the categories in TrashIVL are subdivided.

### 3.1. Sources of TrashIVL

These datasets were originally created using RGB imaging sensors in various real-world environments, capturing images with different backgrounds and lighting conditions. The included datasets are as follows: Drinking waste classification [14], Waste pictures [15], Trashbox [16], TACO [21], Garbage dataset [17], Kaggle garbage classification dataset [18], Acqualtrash [22], Huaweiyun garbage classify learning [19], and Garbage265 [20].

**Figure 1:**
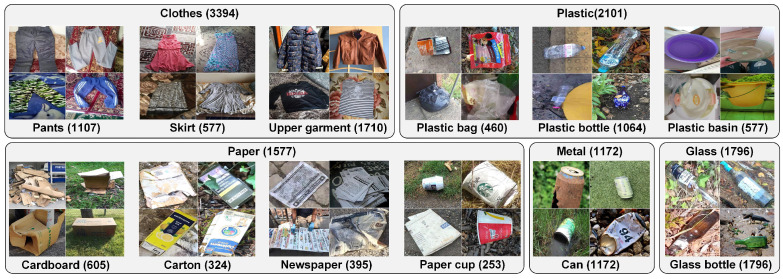
Sample images and category distributions of TrashIVL. TrashIVL consists of five major categories and 12 subcategories. The corresponding sample size is indicated in parentheses after each category.

**Figure 2:**
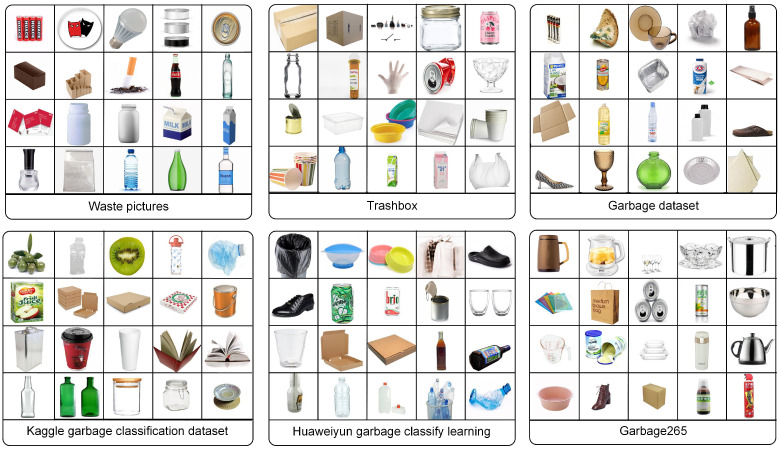
Sample images without backgrounds of public trash datasets that are excluded in TrashIVL.

As shown in Figure 1, we selectively collect images with backgrounds that closely resemble real-life scenarios, such as grassy fields, lose leaves, floors, and roads. Images without background as shown in Figure 2 are screened out. For detection datasets like TACO and Acqualtrash, we select and crop the target garbage boxes for use in TrashIVL. Table 2 shows the corresponding sample distributions of the public datasets that constitute TrashIVL. There are relatively fewer images collected from Acqualtrash, Garbage dataset, and Drinking waste classifications. This is because some of the data in the Acqualtrash overlaps with TACO. Additionally, most of the trash images in the Garbage dataset and Drinking waste classifications do not have backgrounds.

### 3.2. Subdivision of TrashIVL

TrashIVL comprises five classes of recyclable trash (TrashIVL-5): clothes, plastic, paper, metal, and glass. To achieve precise recycling processes and promote resource reuse, we have further subdivided these five major classes into 12 classes (TrashIVL-12). Specifically, we have subdivided clothes into pants, skirt, and upper garment; plastic into plastic bag, plastic bottle, and plastic basin; and paper into cardboard, carton, newspaper, and paper cup. Metals are typically found in the form of cans, while glass primarily exists in the form of glass bottles. They can be recycled multiple times without compromising their quality [23,24]. Given their practical implications, TrashIVL does not make a further subdivision of metal and glass. Figure 1 shows the sample quantities and category distributions. Our dataset is substantial in size and abundant in samples, and thus provides large data support for training deep learning models.

## 4. Proposed Method

In this section, we first introduce the proposed method for recyclable trash sorting, and then describe IPAM by detailing its CAM, SAM, and connectivity.

### 4.1. Establishment of a Recyclable Trash Sorting Network

The specific trash sorting process in this work unfolds as follows: (1) Input the TrashIVL dataset. (2) Perform data augmentation, whereby trash images are uniformly cropped to 224 × 224, randomly flipped, normalized, and rotated up to 15 degrees for improved model robustness. (3) Insert the IPAM into various CNN backbones. (4) Output the corresponding labels of the trash categories in TrashIVL.

**Figure 3:**
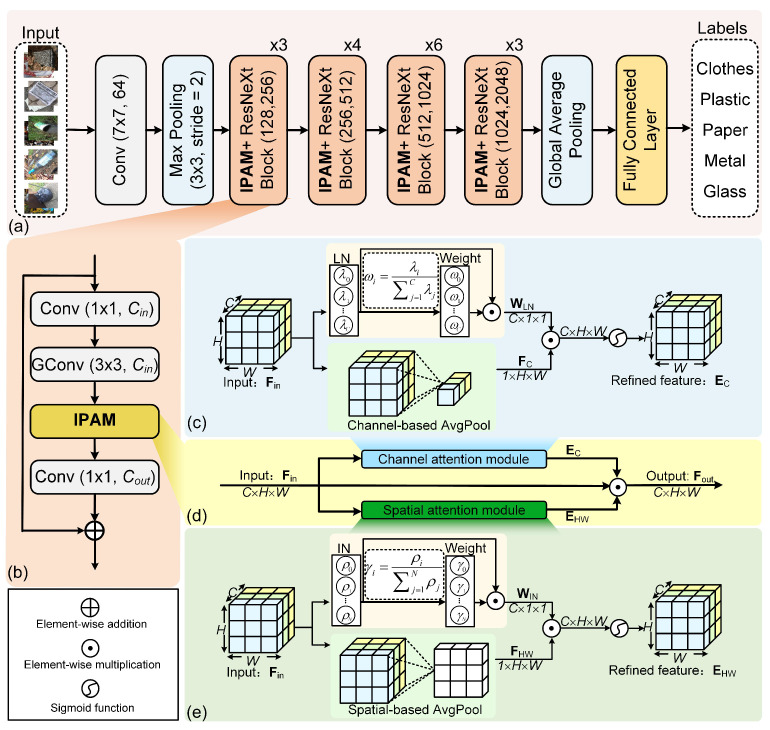
The architecture of recyclable trash sorting. (**a**) The recyclable trash sorting network, (**b**) the structure of the IPAM+ResNeXt block ($C_{\mathrm{in}}$, $C_{\mathrm{out}}$), where $C_{\mathrm{in}}$ and $C_{\mathrm{out}}$ represent the number of convolutional kernels, and GConv denotes group convolution with a group number of 32, (**c**) channel attention module (CAM) of IPAM, (**d**) the structure of IPAM, and (**e**) spatial attention module (SAM) of IPAM.

To improve the accuracy of trash sorting, we select ResNeXt50 [25] as the best backbone through comparison (see Section 5.3 for details) and build a recyclable trash sorting network based on IPAM. The structure of the proposed network is shown in Figure 3a. The ResNeXt block is the core of the ResNeXt modelling component, which enhances feature representation through its multi-branch structure. The structure of the IPAM+ResNeXt block is given in Figure 3b. It starts with a 1×1 convolution layer with $C_{\mathrm{in}}$ kernels. Following this, the 3×3 group convolution serves for feature extraction. The grouping helps to increase the width of the model and thus improves its representation of the features. A 3×3 group convolution extracts the representation of the input features. The insertion of channel–spatial attention after this helps to introduce more information on top of the feature abstraction and to mine and process the features more deeply. Therefore, we insert IPAM after a 3×3 group convolution. Next, the 1×1 convolution raises the channel dimension to $C_{\mathrm{out}}$. Finally, the features learned from each branch are fused together using element-wise addition.

### 4.2. Integrated Parallel Attention Module (IPAM)

Employing an attention module enables the model to highlight specific aspects of the trash, which helps to recognize trash images. Among previous attention modules, the normalization-based attention module (NAM) achieves better accuracy in image classification tasks [12]. Additionally, NAM avoids the addition of convolutional and fully connected layers compared to widely used attention modules such as convolutional block attention module (CBAM) [26] and squeeze-and-excitation (SE) [27]. Nevertheless, we find that there are two problems: (1) It generally adopts a sequential connection. This approach may result in the interference between the spatial and channel attention. (2) It uses batch normalization (BN) to compute the attention weights. During training, BN computes the mean and variance based on the current training batch, which helps normalize the data according to the batch’s specific distribution. However, during the testing phase, the mean and variance used are pre-computed from the training data [28]. Since the distribution of the testing data may differ from that of the training data, these pre-computed values may not accurately represent the characteristics of the testing data.

Taking these issues into account, we have developed the integrated parallel attention module (IPAM). IPAM is a channel–spatial attention module. It parallelizes channel attention with spatial attention, which allows each to independently focus on the channel and spatial information. The structure of IPAM is depicted in Figure 3d. Inspired by NAM, we use adaptive normalization parameters to discern critical information interplay among features. Layer normalization (LN) [29] and instance normalization (IN) [30] are independent of batch dimension. Hence, we establish the channel attention module (CAM) and the spatial attention module (SAM) of IPAM based on LN and IN, respectively.

#### 4.2.1. Channel Attention Module (CAM)

LN normalizes each sample instance, aiming to capture the dependency between different channels. This approach makes the representation of each channel more stable during training and helps accelerate convergence. As shown in Figure 3c, we build the CAM based on LN.

For the input feature map, $F_{\mathrm{in}}\in R^{H\times W\times C}$, where *H*, *W*, and *C* represent its height, width, and number of input channels, respectively. We apply LN to $F_{\mathrm{in}}$ as follows:

(1) $$F_{\mathrm{LN}}≜\mathrm{LN}\left(F_{\mathrm{in}}\right)=\lambda \frac{F_{\mathrm{in}}-\mu }{\sigma }+\varphi ,$$

where μ and σ are the mean and standard deviation of $F_{\mathrm{in}}$, respectively. Both λ and φ are trainable affine transformation factors used to control scaling and shifting operations, continuously adjusted through the learning process of the model. λ serves as the scaling factor from the LN, which is used to gauge the variance of the samples and signify their significance. Greater variance indicates a more diverse sample with abundant information that signifies its greater importance. Conversely, a sample with smaller variance contains less information and is less important.

Then, we compute the the sample-based attention weights as

(2) $$W_{\mathrm{LN}}=\omega ⊙F_{\mathrm{LN}},$$

where ⊙ denotes element-wise multiplication. $\omega =[\omega_{1},\omega_{2},\cdots ,\omega_{C}]$ is an attention vector, in which $\omega_{i}$(1≤i≤C) is computed as

(3) $$\omega_{i}=\frac{\lambda_{i}}{\sum_{j=1}^{C}\lambda_{j}},$$

where $\lambda_{i}$ represents the scaling factor of the *i*-th channel obtained from LN. From Equation (3), ω can be interpreted as a weight value for each feature dimension that signifies the adjustment of the importance of each feature dimension after normalization.

To capture the global channel information, we derive the channel statistics $F_{C}$ through channel-based average pooling:

(4) $$F_{C}=\mathrm{AvgPool}\left(F_{\mathrm{in}}\right).$$

By multiplying $W_{\mathrm{LN}}$ with $F_{C}$ and constraining their product to the range (0, 1) using a sigmoid function, we have

(5) $$E_{C}=\mathrm{Sigmoid}\left(W_{\mathrm{LN}}⊙F_{C}\right).$$

$E_{C}$ can effectively represent the relative channel-based importance.

#### 4.2.2. Spatial Attention Module (SAM)

Instance normalization (IN) performs independently on each channel, assisting the model to focus more on local features at each location. It assesses the importance of different regions based on the local features of the image. This motivates our design of SAM. As shown in Figure 3e, the principle of SAM resembles that of CAM.

For the input feature map $F_{\mathrm{in}}$, IN can be represented as

(6) $$F_{\mathrm{IN}}≜\mathrm{IN}\left(F_{\mathrm{in}}\right)=\rho \frac{F_{\mathrm{in}}-\mu_{ti}}{\sigma_{ti}}+\tau ,$$

where $\mu_{ti}$ and $\sigma_{ti}$ are the mean and standard deviation of the *i*-th channel of the *t*-th sample, respectively. ρ scales the normalized features, enabling the network to learn the significance of specific sample instances. τ introduces an offset to the normalized features so that the model can shift to different sample instances and thereby preserve some aspects of the original feature representation.

The sample instance-based attention weight $W_{\mathrm{IN}}$ is computed as

(7) $$W_{\mathrm{IN}}=\gamma ⊙F_{\mathrm{IN}},$$

where $\gamma =[\gamma_{1},\gamma_{2},\cdots ,\gamma_{N}]$ is an attention vector, whose element is calculated as

(8) $$\gamma_{i}=\frac{\rho_{i}}{\sum_{j=1}^{N}\rho_{j}},$$

where $\rho_{i}$ is the scaling factor of the *i*-th sample. IN normalizes the features along the H×W dimensions, leading to $W_{\mathrm{IN}}$ becoming 1×1 in spatial dimensions, and the number of channels remains as *C*.

Spatial-based average pooling generates global spatial information $F_{\mathrm{HW}}$ as

(9) $$F_{\mathrm{HW}}=\mathrm{AvgPool}\left(F_{\mathrm{in}}\right),$$

and then the relative importance $E_{\mathrm{HW}}$ based on spatial pixel points is computed as

(10) $$E_{\mathrm{HW}}=\mathrm{Sigmoid}\left(W_{\mathrm{IN}}⊙F_{\mathrm{HW}}\right).$$

#### 4.2.3. Parallel Connection

Channel attention and spatial attention, respectively, target the channel and spatial dimensions of the feature map. The parallel connection enables the model to concurrently regulate the feature maps from both the channel and spatial perspectives, which enhances the richness of the feature representations. Consequently, IPAM uses channel attention and spatial attention in parallel to comprehensively learn the relationships between features. The output feature map $F_{\mathrm{out}}$ of IPAM is

(11) $$\begin{array}{c} F_{\mathrm{out}}=E_{C}⊙E_{\mathrm{HW}}⊙F_{\mathrm{in}} \end{array}.$$

## 5. Experiments

### 5.1. Implementation Details

All experiments are conducted using PyTorch 1.10.2 and Python 3.6 on a 3090 GPU. We use the Adam optimizer with a learning rate of 0.00001. After pre-conditioning, the epoch and batch size are set to 80 and 16, respectively. The training and testing set are divided in an 8:2 ratio.

### 5.2. Evaluation Metrics

We apply accuracy, recall, kappa coefficient, precision, and F1-Score to comprehensively evaluate the sorting performance of the models. All evaluation metrics are recorded as (meanvalue±standarddeviation), obtained from the five-fold cross-validation. Accuracy measures the model’s ability to correctly categorize trash. It indicates the percentage of samples correctly predicted by the model out of the total samples:

(12) $$\mathrm{Accuracy}=\frac{\mathrm{TP}+\mathrm{TN}}{\mathrm{TP}+\mathrm{FP}+\mathrm{TN}+\mathrm{FN}},$$

where true positive (TP) denotes the number of positive cases correctly predicted as positive, true negative (TN) represents the number of negative cases correctly predicted as negative, false positive (FP) indicates the number of negative cases incorrectly predicted as positive, and false negative (FN) is the number of positive cases incorrectly predicted as negative.

Recall is the proportion of actual positive cases (trash correctly identified) that the model correctly identified:

(13) $$\mathrm{Recall}=\frac{\mathrm{TP}}{\mathrm{TP}+\mathrm{FN}}.$$

Precision represents the proportion of predicted trash cases that are actually correct:

(14) $$\mathrm{Precision}=\frac{\mathrm{TP}}{\mathrm{TP}+\mathrm{FP}}.$$

F1-Score is the harmonic mean of precision and recall:

(15) $$F1=\frac{2\times \mathrm{Precision}\times \mathrm{Recall}}{\mathrm{Precision}+\mathrm{Recall}}.$$

The kappa coefficient takes into account the consistency between the classification results and the randomized classification results:

(16) $$\mathrm{Kappa}=\frac{\mathrm{Accuracy}-p_{e}}{1-p_{e}},$$

where $p_{e}$ denotes the probability that the classification result randomly matches the true case.

### 5.3. Construction of the Proposed Network

To determine the most suitable backbone and construct our recyclable trash sorting network, we employ several superior pre-tained classification models (ResNet50 [31], EfficientNet-B7 [32], DenseNet121 [33], Xception [34], and ResNeXt50 [25]) as backbones and insert IPAM into them. All these backbones are pre-trained models. Their comparative performance is detailed in Table 3. Notably, ResNeXt50 demonstrates the highest performance. When sorting five classes of recyclable trash, ResNeXt50 achieves an accuracy of 96.25%, a recall of 95.72%, a kappa coefficient of 95.13%, a precision of 95.82%, and an F1-Score of 95.76%. Furthermore, it excels in fine recycling with an accuracy of 92.16%. The unique ability of ResNeXt50 to form multiple branches during the feature learning process proves instrumental. This diversity empowers the network to comprehensively understand and learn the intricacies of the input data, enhancing the capability to characterize complex features in trash sorting tasks. When IPAM is inserted into the backbones, performance improves across all models. For example, on TrashIVL-5, ResNet50 with IPAM shows a slight improvement in accuracy from 96.13% to 96.22% and recall from 95.57% to 96.02%. DenseNet121 experiences a larger boost, with accuracy rising from 96.01% to 97.08% and F1-Score from 94.83% to 96.52%. Xception also sees a significant increase in precision, from 95.75% to 96.90%. The improvement can be attributed to IPAM’s effectiveness in extracting more meaningful features by applying both channel and spatial attention. This dual attention allows models to focus on key features and spatial details simultaneously. Based on the experiment, we select ResNeXt50 as the backbone and insert IPAM into it for constructing our network. ResNeXt50+IPAM not only performs well without IPAM but also shows significant gains with its inclusion, which achieves the best balance of feature representation and classification accuracy for our recyclable trash sorting task.

### 5.4. Arrangement of Channel and Spatial Attention

In both CBAM [26] and NAM [12], channel attention and spatial attention are connected in a serial manner. Initially, channel attention is employed to focus on the channel-based features of the image. Subsequently, the feature map obtained after channel attention is used as input for spatial attention. In contrast, the approach of using channel attention followed by spatial attention (CAM-SAM) offers distinct advantages compared to the serial connection of spatial attention followed by channel attention. This sequential process allows the model to first concentrate on integrating and selecting the features in the channel dimension. It then proceeds to weigh and integrate the features in the spatial dimension. This sequence better aligns with the hierarchical representation of features and the integration process.

Table 4 compares ResNeXt50+IPAM (CAM-SAM in parallel) with the CAM and SAM of IPAM connected serially (CAM-SAM). As shown, the parallel connection yields superior results compared to the serial connection. By employing CAM and SAM in parallel, the model gains a more comprehensive understanding of feature relationships. This diversity may assist the model in better adapting to various types of features, which ultimately enhances the generalization ability of the model.

### 5.5. Performance of Channel Attention and Spatial Attention

To validate the effectiveness of CAM and SAM in IPAM, we use the ResNeXt50 backbone and compare CAM and SAM in CBAM [26], NAM [12], and IPAM on TrashIVL-5 and TrashIVL-12. CBAM is a widely used attention mechanism. It serves as a foundational approach by applying channel–spatial attention. NAM improves upon this by using BN to compute attention weights across both dimensions. Our proposed IPAM builds on NAM by incorporating LN and IN to further refine both channel and spatial attention. While all three methods utilize channel and spatial attention, their internal architectures differ. CBAM follows a more traditional approach, NAM introduces BN, and IPAM enhances the mechanism with LN and IN to improve overall performance. Therefore, we compared the performance of these three attention mechanisms in terms of their channel and spatial attention methods, which helps to assess their respective strengths and improvements. Table 5 shows that CAM in IPAM outperforms both CBAM and NAM in trash sorting on TrashIVL-5 and TrashIVL-12. The improvement in performance can be attributed to LN’s ability to enhance the representation of each channel, allowing the model to better focus on important channel-specific features and accelerating convergence. Similarly, Table 6 demonstrates that SAM in IPAM achieves better results than SAM in CBAM and NAM. The use of IN in IPAM allows SAM to focus more effectively on spatial details, improving the model’s ability to capture relevant spatial information while minimizing interference from less important regions. In conclusion, the experimental results demonstrate that IPAM’s combination of LN and IN enables the model to better capture and process both channel and spatial features, leading to superior performance in trash sorting tasks compared to CBAM and NAM.

### 5.6. Different Attention Modules Inserted into ResNeXt50

**Figure 4:**
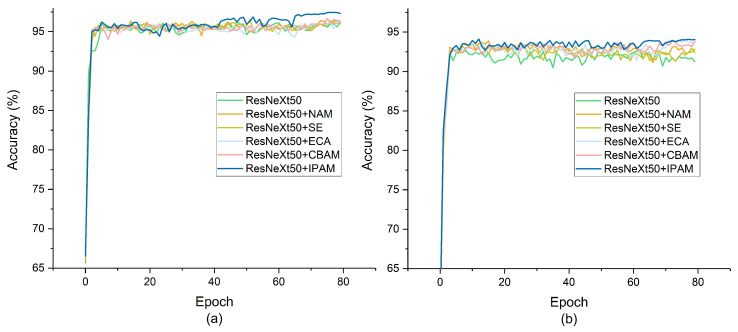
Accuracy curve of different attention modules inserted into ResNeXt50. (**a**) Accuracy curve based on TrashIVL-5. (**b**) Accuracy curve based on TrashIVL-12.

To validate the effectiveness of IPAM, we insert SE [27], efficient channel attention (ECA) [35], CBAM [26], and NAM [12] into ResNeXt50 for recyclable trash sorting and compare them with our proposed recyclable trash sorting network (ResNeXt50+IPAM). The results are presented in Table 7. Since these attention modules are plug-and-play, to ensure a fair comparison, we inserted all of them in the same position as IPAM, as illustrated in Figure 3b. In TrashIVL-5, IPAM improves the sorting ability of ResNeXt50 by 1.17%. As depicted in Figure 4a, the accuracy of ResNeXt50+IPAM stabilizes at around 97% after 65 epochs, surpassing other attention modules. In TrashIVL-12, ResNeXt50+IPAM achieves the finest-grained recycling with a classification accuracy of 94.08%, a recall of 93.69%, a kappa coefficient of 93.80%, a precision of 94.01%, and an F1-Score of 93.32%. As observed in Figure 4b, the accuracy curve of ResNeXt50+IPAM demonstrates greater stability compared to other models. This may be attributed to our special design that the CAM and SAM of IPAM are computed using LN and IN, respectively. LN and IN are independent of batch statistical information, allowing them to maintain normalization even in cases of small batch training or inference. The stable normalization capability contributes to the consistent performance of the recyclable trash sorting network built on IPAM.

**Figure 5:**
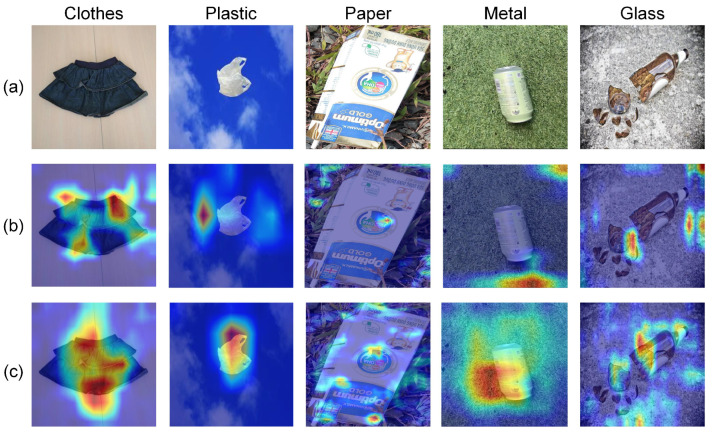
Trash feature maps generated by Grad-CAM. Samples of clothes, plastic, paper, metal, and glass were randomly chosen for illustration. (**a**) Original samples, (**b**) the Grad-CAM images of ResNeXt50, and (**c**) the Grad-CAM images of ResNeXt50+IPAM.

Figure 5 shows the Grad-CAM images using ResNeXt50 and ResNeXt50+IPAM, respectively. Grad-CAM is a technique aiding in comprehending the decision-making process of the network. It generates heat maps illustrating the image regions the neural network prioritizes [36]. These highlighted regions represent the features extracted by the network, signifying the areas of focus during classification. Notably, in scenarios with complex backgrounds such as falling leaves, grass, and sky, these backgrounds can disrupt the classification task, leading ResNeXt50 to concentrate on the background during classification. Nevertheless, with the insertion of IPAM, ResNeXt50+IPAM effectively directs its focus to the trash itself.

### 5.7. Performance of Trash Sorting Models

In previous research, various networks, including AlexNet [6], DenseNet169 [7], EfficientNet-B2 [10], optimized DenseNet121 [8], and AM-b Xception [9], have been employed for trash sorting. We investigate the sorting performance of the proposed recyclable trash sorting network (ResNeXt50+IPAM), compared to the networks used in previous studies. Table 8 lists the results on the public dataset TrashNet and our TrashIVL-5 and TrashIVL-12. Although TrashNet has certain limitations, such as the absence of background in its images, it remains a widely used benchmark dataset for validating trash sorting algorithms in previous studies. By conducting experiments on TrashNet, we ensure a fair and consistent comparison with prior work, allowing us to validate the effectiveness of our proposed method. Deeper networks typically yield more robust feature representations and are adept at learning abstract and complex features. Specifically, DenseNet169 and optimized DenseNet121 are deep and exhibit strong learning capabilities. However, they may face limitations in handling image background interference. In practical applications such as trash sorting, it is essential to consider the influence of image background on the classification models. The insertion of an attention mechanism assists the network in highlighting image regions relevant to the classification task. EfficientNet-B2, AM-b Xception, and ResNeXt50+IPAM all have inserted attention modules. In comparison, our ResNeXt50+IPAM demonstrates superior performance.

**Figure 6:**
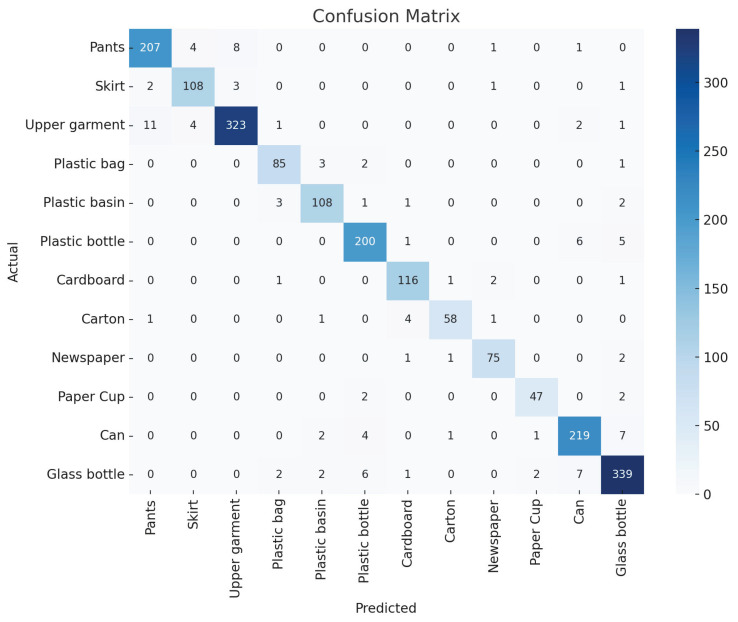
Confusion matrix for ResNeXt50+IPAM based on TrashIVL-12.

The classification performance of models on the TrashIVL-12 dataset is lower compared to the TrashNet and TrashIVL-5 datasets. For further investigation, we present the confusion matrix of ResNeXt50+IPAM on TrashIVL-12 in Figure 6, which highlights areas where the model faces challenges in classification. The model struggles to distinguish between certain materials (e.g., pants, skirts, and upper garments; cardboard and carton; plastic bottles and glass bottles). These items indeed have similar appearances, making classification more difficult. Nevertheless, ResNeXt50+IPAM correctly classifies the majority of objects, demonstrating its overall effectiveness. The ResNeXt50 backbone provides a strong foundation with its multi-branch structure, which increases model width and improves the ability to capture diverse and abstract features. Additionally, IPAM introduces both CAM and SAM, utilizing LN and IN. These techniques allow the model to focus on the most relevant features while filtering out irrelevant information. This channel–spatial attention mechanism is particularly useful in trash sorting tasks, where distinguishing between similar objects and backgrounds is crucial.

## 6. Conclusions

In this work, we have presented a comprehensive approach to automated recyclable trash sorting from a dataset and algorithmic perspective. First, we introduce TrashIVL, which is a dataset comprising five classes of recyclable trash. Images of TrashIVL are collected from public datasets by selectively excluding images without background. To enable fine-grained recycling, TrashIVL can be further subdivided into 12 classes. Then, we introduce the integrated parallel attention module (IPAM) to assist CNNs in reducing interference from the image background. IPAM is a parallel-connected channel–spatial attention. It scales and adjusts normalized features so that the model adaptively learns the importance of different features. After that, we construct the recyclable trash sorting network by inserting IPAM into ResNeXt50. This network achieves a sorting accuracy of 97.42% on TrashIVL-5 and 94.08% on TrashIVL-12, demonstrating its effectiveness in trash sorting.

In the future, we will continue to collect more recyclable trash images to expand TrashIVL. Meanwhile, we will further optimize IPAM and explore its classification effect on other trash datasets.

## Figures and Tables

**Table 1:** Comparison of TrashIVL and other trash image classification datasets. In the “With background” column, × indicates datasets with no background images, ◯ indicates a mixture of images with and without backgrounds, and ✔ indicates datasets where all images have backgrounds.

Trash Image Classification Datasets	Classes	Sub Classes	With Background	Recyclable Trash
TrashNet [13]	6	×	×	✔
Drinking waste classification [14]	4	×	◯	✔
Waste pictures [15]	34	×	◯	×
Trashbox [16]	7	✔	◯	×
Garbage dataset [17]	10	×	◯	×
Kaggle garbage classification dataset [18]	2	×	◯	×
Huaweiyun garbage classify learning [19]	40	×	◯	×
Garbage265 [20]	265	×	◯	×
**TrashIVL (ours)**	**5**	✔	✔	✔

**Table 2:** The corresponding sample distributions of the public datasets constituting TrashIVL.

Dataset	Clothes	Plastic	Paper	Metal	Glass
Drinking waste classification [14]	-	41	-	20	13
Waste pictures [15]	-	41	53	108	154
TACO [21]	-	233	199	-	56
Garbage dataset [17]	-	15	36	23	3
Trashbox [16]	-	319	733	152	505
Kaggle garbage classification dataset [18]	3044	52	486	161	330
Acqualtrash [22]	-	16	7	5	6
Huaweiyun garbage classify learning [19]	116	1092	-	287	729
Garbage256 [20]	234	292	55	416	-

**Table 3:** Comparison of different backbones with and without IPAM for trash sorting.

Datasets	Models	Accuracy (%)	Recall (%)	Kappa (%)	Precision (%)	F1-Score (%)
TrashIVL-5	ResNet50 [31]	96.13 ± 0.18	95.57 ± 0.11	94.97 ± 0.23	95.65 ± 0.21	95.61 ± 0.16
	**ResNet50+IPAM**	**96.22 ± 0.12**	**96.02 ± 0.26**	**95.09 ± 0.18**	**95.68 ± 0.26**	**96.02 ± 0.08**
	EfficientNet-B7 [32]	95.12 ± 0.07	94.40 ± 0.12	94.47 ± 0.05	94.57 ± 0.02	93.55 ± 0.11
	**EfficientNet-B7+IPAM**	**95.35 ± 0.18**	**94.89 ± 0.14**	**94.22 ± 0.23**	**94.65 ± 0.28**	**94.68 ± 0.17**
	DenseNet121 [33]	96.01 ± 0.12	95.52 ± 0.10	95.80 ± 0.15	95.46 ± 0.08	94.83 ± 0.12
	**DenseNet121+IPAM**	**97.08 ± 0.15**	**96.80 ± 0.17**	**96.08 ± 0.19**	**96.55 ± 0.15**	**96.52 ± 0.09**
	Xception [34]	96.24 ± 0.04	95.54 ± 0.04	95.02 ± 0.05	95.75 ± 0.14	95.64 ± 0.07
	**Xception+IPAM**	**97.36 ± 0.11**	**96.82 ± 0.19**	**96.38 ± 0.15**	**96.90 ± 0.20**	**96.85 ± 0.12**
	ResNeXt50 [25]	96.25 ± 0.07	95.72 ± 0.12	95.13 ± 0.09	95.82 ± 0.19	95.76 ± 0.06
	**ResNeXt50+IPAM**	**97.42 ± 0.14**	**96.88 ± 0.09**	**96.36 ± 0.18**	**97.12 ± 0.16**	**96.99 ± 0.11**
TrashIVL-12	ResNet50 [31]	91.62 ± 0.42	90.86 ± 0.42	90.57 ± 0.47	89.58 ± 0.85	90.04 ± 0.61
	**ResNet50+IPAM**	**92.22 ± 0.36**	**91.85 ± 0.39**	**91.28 ± 0.40**	**90.09 ± 0.58**	**91.12 ± 0.48**
	EfficientNet-B7 [32]	91.55 ± 0.08	91.78 ± 0.44	91.54 ± 0.24	90.29 ± 0.10	90.73 ± 0.09
	**EfficientNet-B7+IPAM**	**92.10 ± 0.20**	**92.02 ± 0.18**	**91.89 ± 0.26**	**91.08 ± 0.32**	**91.96 ± 0.29**
	DenseNet121 [33]	90.57 ± 0.45	89.90 ± 0.76	89.99 ± 0.59	88.42 ± 0.62	89.38 ± 0.50
	**DenseNet121+IPAM**	**91.68 ± 0.26**	**90.06 ± 0.39**	**90.28 ± 0.44**	**90.06 ± 0.50**	**90.88 ± 0.38**
	Xception [34]	91.93 ± 0.22	91.12 ± 0.35	91.65 ± 0.28	90.38 ± 0.30	90.15 ± 0.38
	**Xception+IPAM**	**93.20 ± 0.16**	**92.88 ± 0.20**	**92.59 ± 0.28**	**92.02 ± 0.16**	**91.68 ± 0.28**
	ResNeXt50 [25]	92.16 ± 0.47	91.54 ± 0.36	91.17 ± 0.52	90.41 ± 0.72	90.82 ± 0.51
	**ResNeXt50+IPAM**	**94.08 ± 0.11**	**93.69 ± 0.08**	**93.80 ± 0.14**	**94.01 ± 0.22**	**93.32 ± 0.30**

**Table 4:** Connection of channel and spatial attention.

Datasets	Backbone	Connection Arrangement	Accuracy (%)	Recall (%)	Kappa (%)	Precision (%)	F1-Score (%)
TrashIVL-5	ResNeXt50	CAM-SAM	94.50 ± 0.20	93.85 ± 0.35	93.89 ± 0.28	93.96 ± 0.30	92.87 ± 0.22
		**CAM-SAM in parallel**	**97.42 ± 0.14**	**96.88 ± 0.09**	**96.36 ± 0.18**	**97.12 ± 0.16**	**96.99 ± 0.11**
TrashIVL-12	ResNeXt50	CAM-SAM	92.24 ± 0.20	91.15 ± 0.25	91.43 ± 0.26	91.86 ± 0.18	91.25 ± 0.28
		**CAM-SAM in parallel**	**94.08 ± 0.11**	**93.69 ± 0.08**	**93.80 ± 0.14**	**94.01 ± 0.22**	**93.32 ± 0.30**

**Table 5:** Quantitative evaluation of different channel attention methods. The “Attention” column refers to the attention mechanism used (CBAM, NAM, or IPAM), each with its unique channel attention structure. The “Models” column represents the ResNeXt50 model with the respective channel attention module inserted.

Dataset	Attention	Models	Accuracy (%)	Recall (%)	Kappa (%)	Precision (%)	F1-Score (%)
TrashIVL-5	CBAM [26]	ResNeXt50+CAM	96.20 ± 0.12	95.62 ± 0.14	95.06 ± 0.16	95.74 ± 0.14	95.90 ± 0.16
	NAM [12]	ResNeXt50+CAM	96.26 ± 0.06	95.75 ± 0.10	95.14 ± 0.08	95.84 ± 0.07	95.97 ± 0.10
	**IPAM**	**ResNeXt50+CAM**	**96.33 ± 0.15**	**95.84 ± 0.15**	**95.24 ± 0.19**	**95.87 ± 0.18**	**95.92 ± 0.25**
TrashIVL-12	CBAM [26]	ResNeXt50+CAM	93.72 ± 0.15	92.78 ± 0.31	92.92 ± 0.17	92.71 ± 0.29	92.67 ± 0.13
	NAM [12]	ResNeXt50+CAM	93.78 ± 0.14	93.46 ± 0.20	92.99 ± 0.16	92.85 ± 0.12	93.09 ± 0.10
	**IPAM**	**ResNeXt50+CAM**	**93.98 ± 0.13**	**93.39 ± 0.35**	**93.21 ± 0.14**	**93.33 ± 0.32**	**93.43 ± 0.52**

**Table 6:** Quantitative evaluation of different spatial attention methods. The "Attention" column refers to the attention mechanism used, and the "Models" column indicates ResNeXt50 with the respective spatial attention module inserted.

Dataset	Attention	Models	Accuracy (%)	Recall (%)	Kappa (%)	Precision (%)	F1-Score (%)
TrashIVL-5	CBAM [26]	ResNeXt50+SAM	96.12 ± 0.15	95.62 ± 0.17	94.96 ± 0.19	95.63 ± 0.20	95.66 ± 0.25
	NAM [12]	ResNeXt50+SAM	96.28 ± 0.26	95.76 ± 0.34	95.78 ± 0.33	95.83 ± 0.32	95.17 ± 0.33
	**IPAM**	**ResNeXt50+SAM**	**96.31 ± 0.21**	**95.74 ± 0.27**	**95.21 ± 0.27**	**95.83 ± 0.26**	**95.96 ± 0.24**
TrashIVL-12	CBAM [26]	ResNeXt50+SAM	93.71 ± 0.26	93.00 ± 0.38	92.91 ± 0.29	92.71 ± 0.11	92.81 ± 0.23
	NAM [12]	ResNeXt50+SAM	93.98 ± 0.30	93.44 ± 0.46	93.21 ± 0.34	93.28 ± 0.41	93.22 ± 0.41
	**IPAM**	**ResNeXt50+SAM**	**94.01 ± 0.11**	**93.53 ± 0.37**	**93.24 ± 0.11**	**93.40 ± 0.07**	**93.22 ± 0.12**

**Table 7:** Comparison of different attention modules inserted into ResNeXt50.

Dataset	Models	Accuracy (%)	Recall (%)	Kappa (%)	Precision (%)	F1-Score (%)
TrashIVL-5	ResNeXt50	96.25 ± 0.07	95.72 ± 0.12	95.13 ± 0.09	95.82 ± 0.19	95.76 ± 0.06
	ResNeXt50+SE [27]	96.35 ± 0.17	95.87 ± 0.36	95.27 ± 0.22	96.02 ± 0.16	95.84 ± 0.24
	ResNeXt50+ECA [35]	96.27 ± 0.20	95.80 ± 0.27	95.17 ± 0.26	95.79 ± 0.21	95.79 ± 0.24
	ResNeXt50+CBAM [26]	96.26 ± 0.17	95.78 ± 0.25	95.15 ± 0.23	95.82 ± 0.21	95.82 ± 0.22
	ResNeXt50+NAM [12]	96.40 ± 0.15	95.85 ± 0.29	95.33 ± 0.19	96.06 ± 0.08	95.94 ± 0.15
	**ResNeXt50+IPAM**	**97.42 ± 0.14**	**96.88 ± 0.09**	**96.36 ± 0.18**	**97.12 ± 0.16**	**96.99 ± 0.11**
TrashIVL-12	ResNeXt50	92.16 ± 0.47	91.54 ± 0.36	91.17 ± 0.52	90.41 ± 0.72	90.82 ± 0.51
	ResNeXt50+SE [27]	93.78 ± 0.19	93.14 ± 0.42	92.99 ± 0.21	92.86 ± 0.46	92.93 ± 0.26
	ResNeXt50+ECA [35]	94.02 ± 0.14	93.32 ± 0.39	93.25 ± 0.16	93.36 ± 0.25	93.25 ± 0.23
	ResNeXt50+CBAM [26]	93.87 ± 0.24	93.31 ± 0.27	93.09 ± 0.27	93.19 ± 0.28	93.18 ± 0.18
	ResNeXt50+NAM [12]	93.84 ± 0.15	93.21 ± 0.17	93.05 ± 0.17	92.99 ± 0.25	93.04 ± 0.18
	**ResNeXt50+IPAM**	**94.08 ± 0.11**	**93.69 ± 0.08**	**93.80 ± 0.14**	**94.01 ± 0.22**	**93.32 ± 0.30**

**Table 8:** Comparison of trash sorting models.

Datasets	Models	Accuracy (%)	Recall (%)	Kappa (%)	Precision (%)	F1-Score (%)
TrashNet	AlexNet [6]	90.08 ± 0.38	88.65 ± 0.40	87.52 ± 0.28	87.37 ± 0.45	87.81 ± 0.52
	DenseNet169 [7]	95.75 ± 0.29	95.39 ± 0.37	94.77 ± 0.35	95.04 ± 0.33	94.62 ± 0.33
	EfficientNet-B2 [10]	94.13 ± 0.20	93.56 ± 0.36	92.78 ± 0.22	92.88 ± 0.30	93.17 ± 0.26
	Optimized DenseNet121 [8]	94.94 ± 0.22	94.25 ± 0.86	93.77 ± 0.28	93.95 ± 0.50	93.77 ± 0.42
	AM-b Xception [9]	94.57 ± 0.35	94.07 ± 0.34	93.32 ± 0.42	93.63 ± 0.60	93.34 ± 0.85
	**ResNeXt50+IPAM**	**96.05 ± 0.14**	**95.50 ± 0.43**	**94.94 ± 0.26**	**94.50 ± 0.17**	**95.16 ± 0.17**
TrashIVL-5	AlexNet [6]	88.86 ± 0.33	87.16 ± 0.48	87.15 ± 0.39	87.21 ± 0.37	85.56 ± 0.43
	DenseNet169 [7]	96.15 ± 0.18	95.63 ± 0.11	95.76 ± 0.12	95.82 ± 0.25	95.26 ± 0.23
	EfficientNet-B2 [10]	96.22 ± 0.14	95.65 ± 0.06	95.74 ± 0.08	95.85 ± 0.98	95.10 ± 0.18
	Optimized DenseNet121 [8]	93.46 ± 0.91	93.93 ± 1.92	94.42 ± 1.38	94.46 ± 1.11	93.49 ± 1.13
	AM-b Xception [9]	96.10 ± 0.18	95.31 ± 0.19	95.47 ± 0.25	95.66 ± 0.31	94.94 ± 0.23
	**ResNeXt50+IPAM**	**97.42 ± 0.14**	**96.88 ± 0.09**	**96.36 ± 0.18**	**97.12 ± 0.16**	**96.99 ± 0.11**
TrashIVL-12	AlexNet [6]	84.16 ± 0.23	82.11 ± 0.44	82.06 ± 0.37	82.46 ± 0.52	82.14 ± 0.26
	DenseNet169 [7]	91.93 ± 0.26	91.52 ± 0.30	91.19 ± 0.18	90.05 ± 0.28	90.16 ± 0.20
	EfficientNet-B2 [10]	91.91 ± 0.22	91.57 ± 0.25	90.14 ± 0.25	91.04 ± 0.20	90.67 ± 0.26
	Optimized DenseNet121 [8]	92.33 ± 0.35	92.25 ± 0.26	92.27 ± 0.24	91.89 ± 0.23	92.04 ± 0.13
	AM-b Xception [9]	93.98 ± 0.23	92.78 ± 0.38	93.21 ± 0.26	92.91 ± 0.28	93.17 ± 0.18
	**ResNeXt50+IPAM**	**94.08 ± 0.11**	**93.69 ± 0.08**	**93.80 ± 0.14**	**94.01 ± 0.22**	**93.32 ± 0.30**

## Data Availability

Data are available at https://github.com/0815LHL/TrashIVL (accessed on 13 August 2024).

## References

[1] Zhu J., Hu T., Zheng L., Zhou N., Ge H., Hong Z. (2024). YOLOv8-C2f-Faster-EMA: An Improved Underwater Trash Detection Model Based on YOLOv8. Sensors.

[2] Chen Z., Yang J., Chen L., Jiao H. (2022). Garbage classification system based on improved ShuffleNet v2. Resour. Conserv. Recycl..

[3] Shiddiq M., Arief D.S., Fatimah K., Wahyudi D., Mahmudah D.A., Putri D.K.E., Husein I.R., Ningsih S.A. (2023). Plastic and organic waste identification using multispectral imaging. Mater. Today Proc..

[4] Buriboev A.S., Rakhmanov K., Soqiyev T., Choi A.J. (2024). Improving Fire Detection Accuracy through Enhanced Convolutional Neural Networks and Contour Techniques. Sensors.

[5] Zhang X., Sheng Z., Shen H.L. (2022). FocusNet: Classifying better by focusing on confusing classes. Pattern Recognit..

[6] Sultana R., Adams R.D., Yan Y., Yanik P.M., Tanaka M.L. Trash and Recycled Material Identification using Convolutional Neural Networks (CNN). Proceedings of the IEEE SoutheastCon.

[7] Zhang Q., Yang Q., Zhang X., Bao Q., Su J., Liu X. (2021). Waste image classification based on transfer learning and convolutional neural network. Waste Manag..

[8] Mao W.L., Chen W.C., Wang C.T., Lin Y.H. (2021). Recycling waste classification using optimized convolutional neural network. Resour. Conserv. Recycl..

[9] Fan M., Xiao L., He X., Chen Y. Trash Classification Network Based on Attention Mechanism. Proceedings of the International Conference on Advanced Computer Theory and Engineering (ICACTE).

[10] Majchrowska S., Mikołajczyk A., Ferlin M., Klawikowska Z., Plantykow M.A., Kwasigroch A., Majek K. (2022). Deep learning-based waste detection in natural and urban environments. Waste Manag..

[11] Cheng L., He Y., Mao Y., Liu Z., Dang X., Dong Y., Wu L. (2024). Personnel Detection in Dark Aquatic Environments Based on Infrared Thermal Imaging Technology and an Improved YOLOv5s Model. Sensors.

[12] Liu Y., Shao Z., Teng Y., Hoffmann N. NAM: Normalization-based Attention Module. Proceedings of the Conference on Neural Information Processing Systems (NIPS).

[13] Thung G., Yang M. (2016). Classification of Trash for Recyclability Status. https://cs229.stanford.edu/proj2016/report/ThungYang-ClassificationOfTrashForRecyclabilityStatus-report.pdf.

[14] Serezhkin A. (2020). Drinking Waste Classification. https://www.kaggle.com/datasets/arkadiyhacks/drinking-waste-classification.

[15] Wang Z. (2020). Waste Pictures. https://www.kaggle.com/datasets/wangziang/waste-pictures.

[16] Kumsetty N.V., Nekkare A.B., Kamath S. TrashBox: Trash Detection and Classification using Quantum Transfer Learning. Proceedings of the Conference of Open Innovations Association (FRUCT).

[17] Kunwar S. (2023). Garbage Dataset. https://www.kaggle.com/datasets/sumn2u/garbage-classification-v2.

[18] Kaggle (2019). Kaggle Garbage Classification Dataset. https://www.cvmart.net/dataSets/detail/242.

[19] Huaweiyun (2020). Huaweiyun Garbage Classify Learning. https://github.com/x670783915/huaweiyun_garbage_classify__learning/tree/master.

[20] Modelscope (2022). Garbage265. https://modelscope.cn/datasets/tany0699/garbage265/summary.

[21] Proença P.F., Simões P. (2020). TACO: Trash Annotations in Context for Litter Detection. arXiv.

[22] Panwar H., Gupta P., Siddiqui M.K., Morales-Menendez R., Bhardwaj P., Sharma S., Sarker I.H. (2020). AquaVision: Automating the detection of waste in water bodies using deep transfer learning. Case Stud. Chem. Environ. Eng..

[23] Soo V.K., Peeters J.R., Compston P., Doolan M., Duflou J.R. (2019). Economic and Environmental Evaluation of Aluminium Recycling based on a Belgian Case Study. Procedia Manuf..

[24] Good I.D. (1986). New bottles for old: The growth of glass recycling. Endeavour.

[25] Xie S., Girshick R., Dollár P., Tu Z., He K. Aggregated Residual Transformations for Deep Neural Networks. Proceedings of the IEEE Conference on Computer Vision and Pattern Recognition (CVPR).

[26] Woo S., Park J., Lee J.Y., Kweon I.S. CBAM: Convolutional Block Attention Module. Proceedings of the European Conference on Computer Vision (ECCV).

[27] Hu J., Shen L., Albanie S., Sun G., Wu E. (2017). Squeeze-and-Excitation networks. IEEE Trans. Pattern Anal. Mach. Intell..

[28] Wu Y., He K. Group Normalization. Proceedings of the European Conference on Computer Vision (ECCV).

[29] Ba J.L., Kiros J.R., Hinton G.E. Layer Normalization. Proceedings of the International Conference on Learning Representations (ICLR).

[30] Ulyanov D., Vedaldi A., Lempitsky V. (2017). Instance Normalization: The Missing Ingredient for Fast Stylization. arXiv.

[31] He K., Zhang X., Ren S., Sun J. Deep Residual Learning for Image Recognition. Proceedings of the IEEE Conference on Computer Vision and Pattern Recognition (CVPR).

[32] Tan M., Le Q. EfficientNet: Rethinking Model Scaling for Convolutional Neural Networks. Proceedings of the International Conference on Machine Learning (ICML).

[33] Huang G., Liu Z., Van Der Maaten L., Weinberger K.Q. Densely Connected Convolutional Networks. Proceedings of the IEEE Conference on Computer Vision and Pattern Recognition (CVPR).

[34] Chollet F. Xception: Deep Learning with Depthwise Separable Convolutions. Proceedings of the IEEE Conference on Computer Vision and Pattern Recognition (CVPR).

[35] Wang Q., Wu B., Zhu P., Li P., Zuo W., Hu Q. ECA-Net: Efficient channel attention for deep convolutional neural networks. Proceedings of the IEEE Conference on Computer Vision and Pattern Recognition (CVPR).

[36] Selvaraju R.R., Cogswell M., Das A., Vedantam R., Parikh D., Batra D. Grad-CAM: Visual Explanations from Deep Networks via Gradient-Based Localization. Proceedings of the IEEE International Conference on Computer Vision (ICCV).

